# Characterizing South Asian and MENA offspring of Alzheimer’s disease and related dementias patients: the SAMENA cohort design

**DOI:** 10.1002/alz.095577

**Published:** 2025-01-09

**Authors:** Michal S Beeri, Rebecca West, Karen Cai

**Affiliations:** ^1^ Herbert and Jacqueline Krieger Klein Alzheimer’s Research Center at Rutgers Brain Health Institute,, New Brunswick, NJ USA

## Abstract

**Background:**

While people of Middle Eastern/North African (MENA) or South Asian descent constitute a rapidly growing segment of the aging population, they are underrepresented in Alzheimer’s disease and related dementias (ADRD) research. This is concerning because dementia risk factors, both modifiable such as diabetes, and non‐modifiable such as the APOE4 genotype, vary among populations due to environmental and evolutionary differences. Exposure to these risk factors during midlife is particularly crucial, significantly increasing the likelihood of dementia later in life. Midlife is a critical time for ADRD prevention since brain pathology associated with ADRD accumulates decades before cognitive decline becomes noticeable.

**Methods:**

To respond to this gap in knowledge, we have begun developing a longitudinal cohort study which will deeply phenotype middle aged individuals (ages 45‐70 at baseline), from MENA or South Asian backgrounds, who are at high risk of developing ADRD due to a parental family history. The cohort is named SAMENA. Every two years, participants will be assessed for risk and protective factors of ADRD, demographics, social determinants of health, psychiatric factors, physical activity, motor function and sleep. Participants will also undergo brain MRI, and provide blood samples for the assessment of existing and novel biomarkers as well as for genetics.

**Results:**

The SAMENA study has recently commenced. By the time of the AAIC 2024 conference, we expect to have enrolled the first 50 participants. At that point, we will present demographic data, the most successful recruitment methods, eligibility/ineligibility statistics, and notable disparities in enrollment patterns.

**Conclusion:**

This study aims to address a critical gap in knowledge in the epidemiology of ADRD and cognitive decline in south Asians and MENA populations, who are largely underrepresented in ADRD research. Identifying factors that contribute to ADRD risk in high‐risk individuals already in midlife may lead to earlier and more targeted prevention and intervention strategies specifically relevant to these populations.

